# Reliability of Handheld Ultrasound Assessment of Brachial Artery Flow-Mediated Dilation Using AI-Assisted Automated Analysis in Postmenopausal Women

**DOI:** 10.3390/medicina62010181

**Published:** 2026-01-15

**Authors:** Wei-Di Chen, Yung-Chia Kao, Chun-Hsien Chiu, Chao-Chun Huang, Mei-Wun Tsai

**Affiliations:** 1 Department of Physical Therapy and Assistive Technology, National Yang Ming Chiao Tung University, Taipei City 112304, Taiwan; wdchen.be11@nycu.edu.tw (W.-D.C.); jacky.be12@nycu.edu.tw (Y.-C.K.); sky1218@nycu.edu.tw (C.-H.C.);; 2 Department of Physical Medicine & Rehabilitation, Far Eastern Memorial Hospital, New Taipei City 22000, Taiwan

**Keywords:** YOLO_v12_, deep learning, flow mediated dilation, vascular endothelium, ultrasound, postmenopausal women

## Abstract

*Background and Objectives:* Endothelial dysfunction is an early indicator of cardiovascular disease and is commonly assessed using flow-mediated dilation (FMD). Although handheld ultrasound (HHUS) devices improve measurement accessibility, image analysis for conventional flow-mediated dilation (FMD) assessment remains time-consuming and highly operator-dependent. This study aimed to evaluate the between-day test–retest reliability of an AI-assisted brachial artery image analysis workflow integrating HHUS imaging with a YOLO_v12_ deep learning model in postmenopausal women. *Materials and Methods:* Seventeen postmenopausal women aged 55–70 years completed two flow-mediated dilation assessments conducted seven days apart. Brachial artery images were acquired using a standardized FMD protocol with a handheld ultrasound system. An AI-assisted image analysis workflow based on a YOLO_v12_ deep learning model was used to automatically measure baseline diameter (D_base_), peak diameter (D_peak_), absolute FMD (FMD_abs_), and relative FMD (FMD%). Between-day reliability was evaluated using intraclass correlation coefficients (ICCs), coefficients of variation (CVs), and Bland–Altman analysis. *Results:* Good between-day repeatability was observed for baseline and peak diameters, with ICCs of 0.81 and 0.76 and low CVs (3.26% and 3.22%), respectively. Functional vascular outcomes also demonstrated good reliability, with ICCs of 0.81 for FMD_abs_ and 0.87 for FMD%. However, higher CVs were observed for FMD_abs_ (17.15%) and FMD% (19.09%), indicating substantial inter-individual variability. Bland–Altman analysis showed a small mean difference for FMD% (0.34%), with no evidence of systematic bias. *Conclusions:* An AI-assisted HHUS image analysis workflow integrating a YOLO_v12_ deep learning model demonstrates acceptable between-day reliability for diameter-based and dilation-based measures of flow-mediated dilation in postmenopausal women. While variability in functional responses exists, the proposed system is feasible for research-oriented vascular assessment, providing a methodological foundation for future validation and clinical translation studies.

## 1. Introduction

Endothelial dysfunction is a key early marker in the development of cardiovascular disease (CVD) and is widely recognized as the initial stage of atherosclerosis [1,2]. In postmenopausal women, estrogen deficiency and age-related vascular changes contribute to impaired endothelial function, increasing the risk of CVD and all-cause mortality [3,4]. Flow-mediated dilation (FMD), assessed using high-resolution B-mode ultrasound, is the gold-standard non-invasive method for evaluating nitric oxide-mediated, endothelium-dependent vasodilation (EDVD) [5,6,7,8,9,10]. Although FMD is a validated and widely studied technique, its routine clinical use remains limited. Conventional FMD assessments are constrained by high equipment costs, technical complexity, the need for trained personnel, and strict imaging conditions [11,12]. Handheld ultrasound (HHUS) devices offer an appealing alternative due to their portability, affordability, and ease of use [13,14,15]. Previous studies have demonstrated that HHUS can produce valid and reproducible measurements of vascular parameters [14], including FMD [16], with performance comparable to that of standard high-end ultrasound systems [16,17,18,19]. However, most current HHUS workflows still rely on manual or semi-automated post-processing of images, which is time-intensive and introduces variability due to operator skill and subjective interpretation [9,20,21].

Recent progress in artificial intelligence (AI), particularly in deep learning, has shown promise in automating medical image analysis, including applications in cardiovascular assessment [22,23,24,25,26], visceral adiposity estimation [27,28], and carotid intima–media thickness measurement [17,29,30]. The YOLO (You Only Look Once) family of models has emerged as a state-of-the-art object detection framework, offering real-time image analysis capabilities [31,32,33]. The latest version, YOLO_v12_, integrates several architectural improvements—such as the Area Attention Module (A^2^) [34], Residual Efficient Layer Aggregation Networks (R-ELAN), and Flash Attention—that enhance detection accuracy and computational efficiency, particularly in identifying fine anatomical structures [35]. Although the reliability of vascular function assessment methods has been established in younger populations, the measurement stability of these methods in healthy middle-aged and older adults, particularly postmenopausal women, remains to be confirmed. The present study, therefore, focuses on evaluating the test–retest consistency of this system in this population, serving as a fundamental methodological step before further application-oriented research. Accordingly, the present study proposes an automated brachial artery image analysis workflow that integrates handheld ultrasound imaging with a YOLO_v12_-based deep learning model. Under standardized measurement conditions, this study aimed to conduct an initial evaluation of the measurement stability and repeatability of the proposed AI-assisted system, providing a methodological foundation for future investigations focused on measurement accuracy, criterion validity, and clinical applicability.

## 2. Materials and Methods

### 2.1. AI-Assisted Image Analysis Workflow

**Figure 1:**
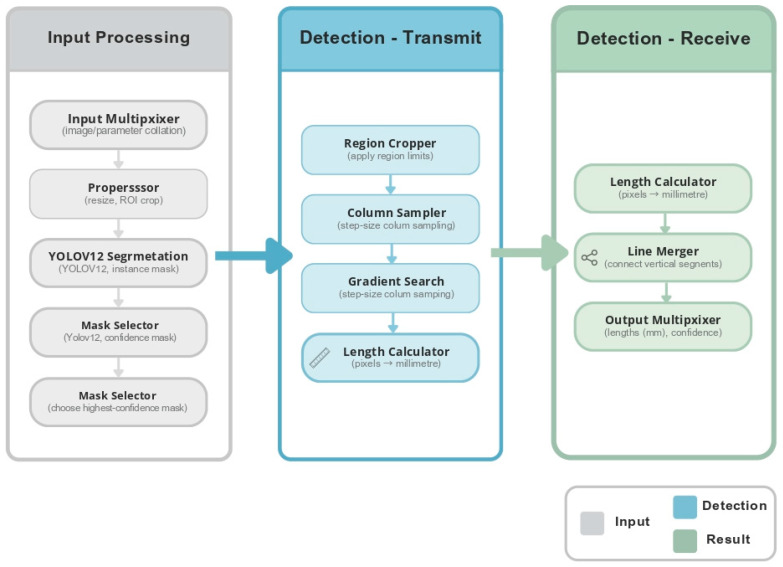
The flow chart of the AI prediction system.

To improve the efficiency and objectivity of vascular image analysis, this study developed an AI-assisted image analysis model based on the YOLO_v12_ architecture for automated detection and measurement of brachial artery diameter in handheld ultrasound (HHUS) images (Figure 1). All ultrasound images were acquired following a standardized flow-mediated dilation (FMD) protocol using a portable HHUS device, with key frames extracted at baseline and peak vasodilation, typically 60–75 s after cuff release.

During model development, 360 images were retrospectively collected as the training dataset, and an additional 40 images were reserved as an independent test dataset. All images were manually annotated by a single experienced ultrasound technician, who delineated the lumen–intima boundaries as the reference standard. The YOLO_v12_ model was trained to automatically detect arterial boundaries and compute vessel diameters across the cardiac cycle. Model training and internal validation used a training/validation split, and final performance was evaluated on the fully independent test dataset [36,37]. All training and test images were completely independent from the images used in the reliability study, ensuring no overlap between model development and reliability testing datasets.

After training, the model was integrated into a semi-automated image analysis workflow, allowing operators to upload HHUS images and obtain automated vessel diameter measurements within seconds. Flow-mediated dilation (FMD%) was defined as the relative change between baseline diameter and peak diameter. This workflow reduces the need for manual frame selection and measurements, thereby decreasing operator dependence and shortening analysis time. However, because the model was trained and validated using retrospectively annotated data from a single institution, its generalizability to external datasets and different ultrasound systems requires further evaluation. The system was primarily designed to verify the feasibility of AI-assisted HHUS image analysis workflows and to support research applications, rather than for clinical diagnostic purposes [19].

### 2.2. Web-Based Platform for Real-Time Image Analysis

**Figure 2:**
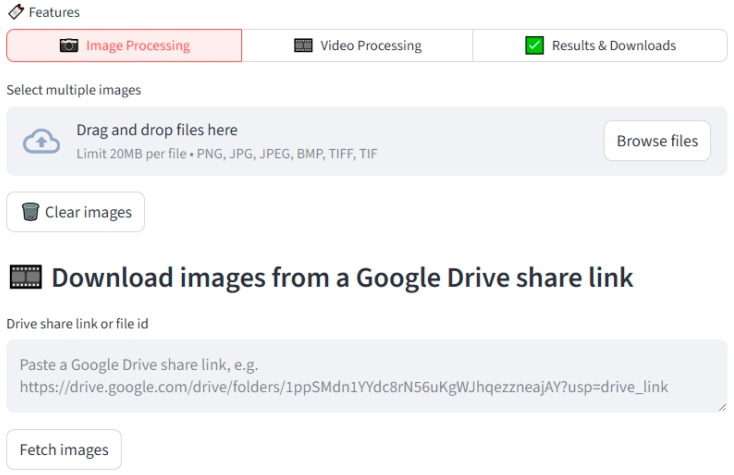
A schematic illustration of the AI-assisted handheld ultrasound (HHUS) vascular image analysis web interface before image upload.

To further enhance the usability of the HHUS image analysis workflow, a web-based platform was developed, as shown in (Figure 2), enabling researchers to upload HHUS images and perform automated brachial artery diameter measurements. The platform integrates the YOLO_v12_ model into the backend computational architecture, with a user interface designed for simplicity and ease of use. Both single-image and batch processing are supported, and analysis results—including brachial artery diameter and corresponding FMD% values—are returned within a short processing time.

**Figure 3:**
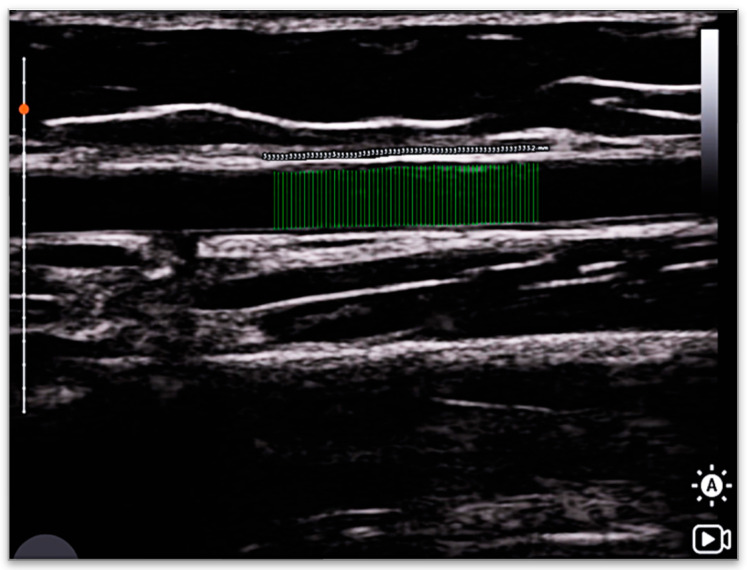
Representative output of the AI-assisted handheld ultrasound (handheld ultrasound, HHUS) vascular image analysis system, showing the results displayed after image upload and completion of the automated analysis.

The backend of the platform employs a standardized server and database architecture to ensure efficient image processing and to meet basic data security requirements. The AI modeling process is executed on the server side, and images of the analysis result are returned to the user, as illustrated in (Figure 3). In its current state, the platform primarily serves as a research tool for deep learning-based vascular image analysis, aiming to provide a reproducible and efficient image processing pipeline to support methodological validation and data analysis.

### 2.3. Participants

**Figure 4:**
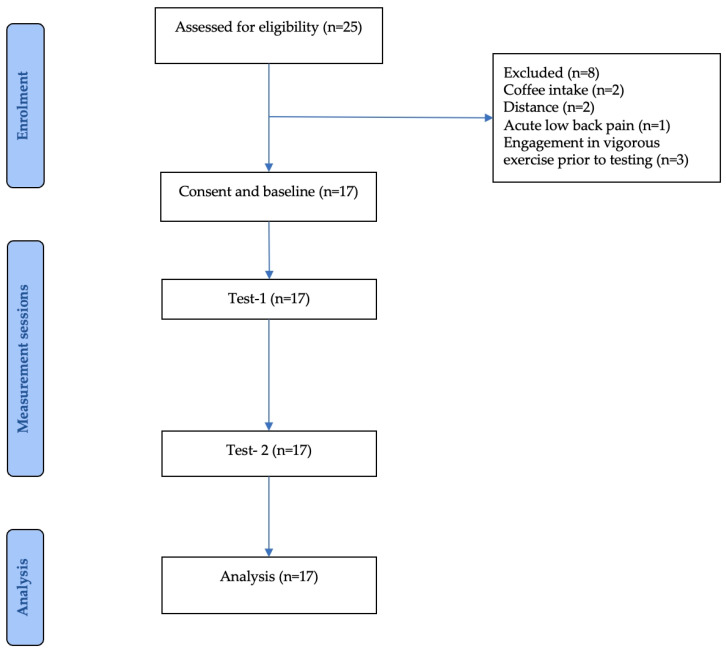
A flow diagram of progress through the phases of a trial.

Seventeen postmenopausal women, aged 55–70 years, were recruited as participants of this study from community settings in Taipei, Taiwan, through telephone interviews, social media announcements, and poster advertisements. The inclusion criteria were met by all participants and were as follows: at least one year post menopause, body mass index (BMI) below 30 kg/m^2^, able to live independently, and able to communicate in Mandarin or Taiwanese. Exclusion criteria included acute musculoskeletal injuries within the past month (e.g., acute inflammation, fracture, sprain, contusion, joint replacement), diagnosed central or peripheral nervous system disorders that may affect exercise participation (e.g., stroke, dementia, Parkinson’s disease, autonomic dysfunction), major cardiovascular events or surgeries within the past six months (e.g., cardiac catheterization with stenting, pacemaker implantation, peripheral or cerebrovascular reconstruction), endocrine disorders (e.g., hyperthyroidism), connective tissue diseases (e.g., scleroderma), peripheral vascular diseases (e.g., peripheral artery disease or venous thrombosis), current or recent use (within six months) of hormone therapy or endocrine-related medications, current use of β-blockers, anti-inflammatory drugs, or anticoagulants, uncontrolled hypertension, and participation in any exercise training programs. Inter-day test–retest reliability was assessed on two separate occasions at the same time of day in the morning to control for circadian influences, with a seven-day interval between sessions, and all measurements were performed during daylight hours. All participants gave written informed consent after receiving a detailed explanation of the study’s purpose, procedures, potential risks, and benefits (Figure 4).

### 2.4. Brachial Artery FMD Procedure

Each participant completed two testing sessions (Test-1 and Test-2) seven days apart, conducted in a quiet laboratory with soft lighting and a controlled room temperature of 23–25 °C. To minimize circadian influences, both sessions were performed at the same time of day. Before testing, participants refrained from vasoactive medications, alcohol, caffeine, stimulants, and vigorous physical activity for 24 h, fasted for ≥6 h, and ensured adequate sleep the night before. Upon arrival, they rested supine for 15–20 min [9]. All measurements were performed by the same trained ultrasound operator to ensure procedural consistency and measurement reliability. Brachial artery imaging was performed on the left arm using a high-resolution handheld ultrasound system (LeSONO LU700 L, Leltek, Inc., New Taipei City, Taiwan) [36]. The ultrasound was operated with the following settings: a frequency of 10 MHz, a depth of 3.2 cm, and a dynamic range of 50–55 dB. Pulse-wave Doppler was applied at an insonation angle of 60°. The probe was positioned 5–10 cm proximal to the antecubital fossa and secured with a magnetic holder. An occlusion cuff (Spirit sphygmomanometer aneroid, CK-112P, Taipei, Taiwan [37]) was placed 5 cm distal to the antecubital fossa and inflated to 200 mmHg (at least 50 mmHg above the individual systolic blood pressure) for 5 min. All images were stored as video files for subsequent offline analysis [9].

### 2.5. Offline Data Analysis

**Figure 5:**
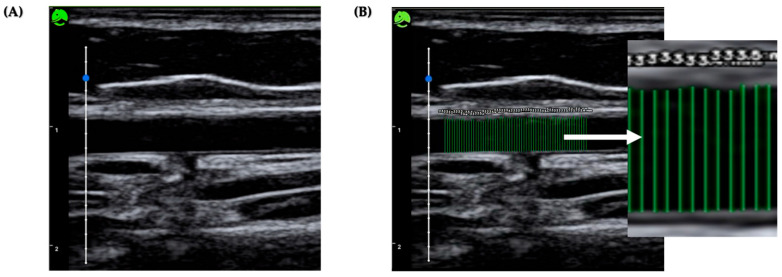
(**A**) A longitudinal-view image of the brachial artery for offline analysis. (**B**) The mean intima-to-intima vertical distance of the brachial artery calculated using a deep learning model. All image analyses were performed using the same standards, as described in the study methodology.

The entire vascular assessment was continuously recorded using screen-capture software, with a total recording duration of approximately 9 min. This included 1 min of baseline recording, 5 min during arterial occlusion, and 3 min of continuous longitudinal imaging following cuff deflation. Ultrasound recordings were analyzed offline using a YOLO_v12_ deep learning model, which automatically detected the intima-to-intima boundaries of the brachial artery and extracted vessel diameters across multiple vertical scan lines per frame. To ensure consistency and adequate image quality for model analysis, frame selection was performed by a trained ultrasound assessor who extracted representative frames from the continuous recordings based on predefined criteria, including clear visualization of the arterial walls, stable probe positioning, and minimal image noise. For each selected frame, the YOLO_v12_ model extracted arterial boundaries along multiple vertical scan lines and generated an average vessel diameter value. Baseline diameter (D_base_) was defined as the arterial diameter during the 30 s period preceding cuff inflation. Within this interval, five representative frames were selected from the continuous recording and individually uploaded to the analysis platform for automated measurement. Each frame yielded a single average diameter value, and the mean of these five values was used to represent D_base_ for that test session. Peak diameter (D_peak_) was defined as the maximum arterial diameter observed within the 60–75 s time window following cuff release after 5 min of occlusion. Similarly, five frames were selected within this window, processed individually by the platform, and the largest of the resulting average diameter values was recorded as D_peak_. Absolute FMD (FMDabs) was calculated as D_peak_ − D_base_, and percent FMD (FMD%) as [(D_peak_ − D_base_)/D_base_] × 100 [7,8,9]. To minimize subjective variability and maintain consistency in image evaluation, all automated measurement outputs were reviewed by a single experienced vascular sonographer. This review ensured appropriate identification of arterial boundaries and adherence to predefined image quality criteria. The described workflow generated reliable arterial diameter measurements for each test session and was used for subsequent test–retest reliability analyses (Figure 5).

### 2.6. Statistical Analysis

All statistical analyses were performed using SPSS Statistics version 29.0 (IBM Corp., Armonk, NY, USA). Continuous variables are presented as the mean ± standard deviation (SD), and categorical variables are presented as percentages. Between-day reliability of handheld ultrasound-derived brachial artery flow-mediated dilation (FMD) measurements was assessed using the intraclass correlation coefficient (ICC) with a two-way random-effects model (ICC [1,3], consistency type), along with corresponding 95% confidence intervals (CIs). The primary outcome variables included baseline diameter (D_base_), peak diameter (D_peak_), and percentage of flow-mediated dilation (FMD%). The coefficient of variation (CV) was also calculated, with values ranging from 0 to 10% indicating excellent variability, 10–20% indicating good variability, 20–30% indicating moderate variability, and values greater than 30% indicating poor variability [38]. The interpretation of ICC values was as follows: ICC < 0.50 indicates poor reliability, 0.50–0.74 indicates moderate reliability, 0.75–0.90 indicates good reliability, and ICC > 0.90 indicates excellent reliability [39]. The coefficient of variation (CV) was calculated as the ratio of the standard deviation to the mean and expressed as a percentage using the following formula [40]:

$$\mathrm{CV}\;(\%)\;=\;\frac{SD}{Mean}\times \;100$$

The standard error of measurement (SEM) was calculated to estimate within-subject measurement error using the following formula [41]:

$$\mathrm{SEM}\;=\;\mathrm{SD}\;\times \sqrt{1-ICC}$$

The minimal detectable change at the 95% confidence level (MDC_95_) was calculated to determine the smallest change exceeding measurement error using the following formula [42]:

$${\mathrm{MDC}}_{95}\;=\;1.96\times \sqrt{2}\;\times \;\mathrm{SEM}$$

Sample size estimation was conducted a priori using a precision-based approach for ICC estimation. Based on a between-day test–retest design with two repeated measurements per participant (k = 2), a 95% confidence level, and the primary reliability outcome defined as flow-mediated dilation (FMD) quantified by percentage diameter change following reactive hyperemia, the expected intraclass correlation coefficient (ICC) was set at 0.8, with the target precision defined as a half-width of ±0.15 for the 95% confidence interval of the ICC estimate. An anticipated dropout rate of 10% was incorporated into sample size planning. Accordingly, a target sample size of 27 participants was estimated to yield an analyzable sample size consistent with the estimated requirement for the desired ICC precision [43,44].

## 3. Results

The study was conducted between April and May 2025. All experimental procedures were performed at the Cardiopulmonary Rehabilitation Laboratory, National Yang Ming Chiao Tung University. After participant screening, a total of 17 participants were included in the study. All participants completed two repeated measurement sessions for subsequent test–retest reliability analysis (Table 1).

### Between-Day Reproducibility of Brachial Artery Diameters and FMD Responses

**Figure 6:**
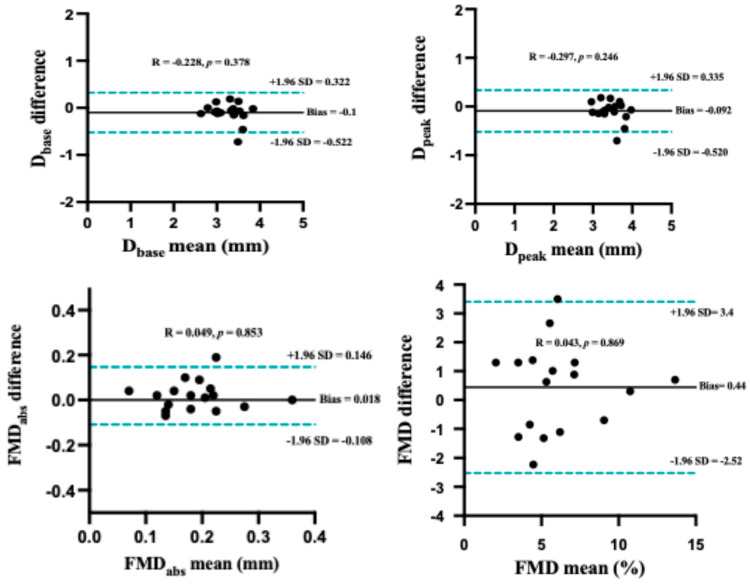
Bland–Altman plots assessing intra-rater agreement for between-day repeated measurements of flow-mediated dilation (FMD). The *Y*-axis represents the difference between Test-1 and Test-2. The solid black line indicates the mean bias, while the dashed gray lines denote the 95% limits of agreement (LoA). No proportional bias was detected, as indicated by the non-significant correlation between the differences and the mean values (*p* > 0.05).

Each participant underwent two flow-mediated dilation (FMD) assessments performed 7 days apart to evaluate inter-day repeatability. The results are summarized in Table 2. The intraclass correlation coefficients (ICCs) for baseline diameter (D_base_) and peak diameter (D_peak_) were 0.81 and 0.76, with low coefficients of variation (CVs) of 3.26% and 3.22%, indicating good repeatability of diameter measurements across days. Hemodynamic parameters demonstrated moderate repeatability, with ICC values of 0.65 for baseline mean blood velocity (MBV) and 0.67 for blood flow (BF). For functional vascular responses, absolute flow-mediated dilation (FMD_abs_) showed an ICC of 0.81 with a CV of 17.15%, indicating good agreement between repeated measurements. The primary outcome measure, relative flow-mediated dilation (FMD%), demonstrated an ICC of 0.87, corresponding to good inter-day reliability, although accompanied by a higher CV of 19.09% (Table 2). This finding suggests that while relative FMD measures are reproducible at the group level, greater within-subject variability may be present. Bland–Altman analysis further supported the repeatability of FMD%, revealing a small mean difference of 0.34% between the two measurement sessions, with 95% limits of agreement (LoA) ranging from −0.25% to 3.40%. The absence of systematic bias across the measurement range indicates acceptable agreement between sessions (Figure 6).

## 4. Discussion

To our knowledge, this study represents the first feasibility and reliability investigation applying a YOLO-based deep learning model for the assessment of flow-mediated dilation. The primary aim was to establish measurement stability and between-day consistency as a methodological foundation for future clinical translation and further validation. Considering the application characteristics of artificial intelligence-assisted image analysis systems, the present study was designed as a reliability-only feasibility study to evaluate the between-day repeatability of flow-mediated dilation (FMD) assessed using a handheld ultrasound system combined with a YOLO_v12_ deep learning-based analysis workflow. Under this study design, the following results provide empirical evidence regarding the between-day measurement performance of the proposed workflow. This study demonstrated that handheld ultrasound (HHUS) integrated with a YOLO_v12_ deep learning image analysis model can achieve good between-day repeatability in the assessment of flow-mediated dilation (FMD). The intraclass correlation coefficients (ICCs) for baseline diameter (D_base_) and peak diameter (D_peak_) were 0.81 and 0.76, respectively, with coefficients of variation (CVs) of 3.26% and 3.22%, indicating stable diameter measurements across days. The ICC values for percent FMD (FMD%) and absolute FMD (FMDabs) were 0.87 and 0.81, respectively, also reflecting good reliability; however, the CVs were relatively high at 19.09% and 17.15%, suggesting substantial inter-individual variation in vascular dilation responses. Notably, the peak post-deflation diameter was observed predominantly within 60–75 s, consistent with previously reported ranges in high-resolution ultrasound studies [45], indicating that our measurement protocol effectively captured the typical time course of vascular response. This study was designed and conducted following established expert consensus guidelines for FMD assessment, including standardized participant preparation, cuff placement, occlusion duration, and post-deflation imaging time frames [7,9,46]. The coefficient of variation (CV) of approximately 19% observed for flow-mediated dilation (FMD) in the present study may reduce sensitivity to detect small intervention-related changes. The present study did not include a direct comparison with high-resolution laboratory-based ultrasound systems, which are still regarded as the gold standard for flow-mediated dilation (FMD) assessment. We fully acknowledge that good reliability alone does not imply measurement accuracy or validity, as a measurement system may demonstrate high repeatability while still exhibiting systematic bias. Despite these limitations, the present study was intentionally designed as a reliability-only feasibility investigation. Whereas previous reliability studies have predominantly focused on younger [47,48] and middle-aged adults [49], the inclusion of postmenopausal women enhances the methodological relevance of the findings for populations that are more representative of future clinical and applied research settings. Establishing measurement stability under such conditions constitutes a necessary methodological step before further validation and clinical translation [50]. From an application perspective, this suggests that the proposed YOLO-based system may be more suitable for cross-sectional screening or population-level assessments rather than for longitudinal monitoring of subtle within-subject changes. The AI-assisted image analysis workflow used in this study has been granted an invention patent by the Taiwan Intellectual Property Office (Patent No. I907279). The patented content primarily pertains to the engineering implementation of the image analysis pipeline and the automated vessel measurement methodology. The purpose of this patent is to establish a clearly defined and reproducible technical framework, facilitating methodological standardization and supporting the structured advancement of subsequent technical validation studies. By explicitly defining the system architecture, data processing workflow, and automated measurement logic, patent protection may help reduce methodological variability across different research settings and implementation environments, thereby providing a consistent engineering foundation for future large-scale or multicenter investigations. Nevertheless, the primary focus of the present study remains on evaluating the feasibility and test–retest reliability of the proposed image analysis workflow. The existence of patent protection does not imply clinical readiness or commercial maturity of the system. Before considering broader translational or applied use, further validation by independent research groups, testing on external datasets, and performance evaluation across different ultrasound platforms are required. Several methodological limitations should be noted. As the handheld ultrasound system used in the present study was unable to simultaneously assess blood velocity and arterial diameter, shear rate and its associated area under the curve could not be calculated. Consequently, the reported outcomes are limited to diameter-based measures of flow-mediated dilation rather than fully shear rate-normalized FMD indices. Accordingly, the physiological interpretation of reactive hyperemia-induced dilation should be made with caution, as diameter-based responses may not fully reflect endothelial function [51]. In addition, vascular reactivity and flow-related measures are inherently sensitive to short-term physiological fluctuations. Despite the use of standardized testing conditions and adequate pre-test rest, some degree of between-day variability remains unavoidable [52]. As each assessment was performed only once per visit, acute psychological or physiological factors present at the time of measurement—such as subjective anxiety reported by participants—may have transiently influenced autonomic balance and vascular tone [53]. These acute effects may therefore contribute to between-day variability in vascular reactivity measures at the measurement level, rather than reflecting true alterations in underlying vascular endothelial function [54,55]. Furthermore, the present study was conducted as an exploratory feasibility and reliability investigation, and the sample size included may be insufficient to yield highly precise intraclass correlation coefficient (ICC) estimates with narrow confidence intervals. Taken together, these considerations emphasize the necessity of interpreting the present findings strictly within the predefined methodological scope of a feasibility and reliability study. Moreover, the findings should be interpreted within the context of a controlled proof-of-concept cohort and should not be extrapolated to male participants, individuals with obesity, or populations with cardiometabolic diseases. Accordingly, the results provide preliminary information on the measurement behavior of an AI-assisted handheld ultrasound workflow in postmenopausal women, thereby establishing a methodological foundation for future system development.

## 5. Conclusions

Our study demonstrates that a handheld ultrasound system integrated with a YOLO_v12_ deep learning model provides consistent between-day measurements of flow-mediated dilation (FMD) in postmenopausal women. Acceptable repeatability is observed for diameter-based outcomes, including baseline and peak diameters, as well as for both absolute and relative measures of FMD. This study is designed solely as a reliability-focused feasibility investigation and does not aim to establish measurement accuracy or clinical validity. Accordingly, the present findings represent preliminary observations describing the repeatability characteristics of the proposed measurement workflow. These findings establish preliminary evidence of system robustness that supports future investigations of validity-oriented applications in postmenopausal women.

## 6. Patents

The invention described in this study has been granted a patent by the Taiwan Intellectual Property Office (TIPO), under Patent No. I907279.

## Figures and Tables

**Table 1:** Demographic characteristics of participants (n = 17).

Variables	Mean ± SD
Age, years	62.6 ± 4.1
Height, m	1.56 ± 0.04
Weight, kg	56.2 ± 5.3
BMI, kg/m^2^	22.8 ± 1.9
Years post menopause, y	8.9 ± 5.3
Heart rate, bpm	65.2 ± 8.1
SBP, mmHg	117 ± 12.6
DBP, mmHg	75.1 ± 5.6
MAP, mmHg	87.7 ± 7

Data are mean ± SD. Abbreviations: BMI: body mass index (kg/m^2^); SBP: systolic blood pressure; DBP: diastolic blood pressure; MAP: mean blood pressure.

**Table 2:** Descriptive statistics and test–retest reliability of flow-mediated dilation parameters.

Variables	Test-1	Test-2	ICC (95% CI)	CV (%)	SEM	MDC_95_
D_base_ (mm)	3.21 ± 0.32	3.31 ± 0.37	0.81 (0.54, 0.92)	3.26	0.31	0.85
D_peak_ (mm)	3.41 ± 0.28	3.5 ± 0.35	0.76 (0.45, 0.9)	3.22	0.15	0.42
MBV (cm/s)	7.92 ± 2.45	8.98 ± 2.58	0.65 (0.27, 0.86)	14.28	0.65	1.82
BF (ml/min)	38.21 ± 13.22	46.98 ± 16.82	0.67 (0.29, 0.86)	18.2	4.64	12.86
FMD_abs_ (mm)	0.2 ± 0.08	0.19 ± 0.08	0.81 (0.54, 0.92)	17.15	0.03	0.08
FMD (%)	6.36 ± 3	5.91 ± 2.93	0.87 (0.67, 0.95)	19.09	1.05	2.92

Data are mean ± SD. Abbreviations: CI: confidence interval; CV: coefficient of variation; D_base_: baseline diameter; D_peak_: peak diameter; FMD_abs_: absolute flow-mediated dilation; FMD: flow-mediated dilation; BF: blood flow; ICC: intraclass correlation coefficient; MBV: mean blood velocity; MDC_95_: minimal detectable change; SEM: standard error of measurement.

## Data Availability

The data presented in this study are available upon reasonable request from the corresponding author. The data are not publicly available due to privacy and ethical restrictions concerning participant information.
